# Proteomics Profiling of Osteoporosis and Osteopenia Patients and Associated Network Analysis

**DOI:** 10.3390/ijms231710200

**Published:** 2022-09-05

**Authors:** Mysoon M. Al-Ansari, Shereen M. Aleidi, Afshan Masood, Eman A. Alnehmi, Mai Abdel Jabar, Maha Almogren, Mohammed Alshaker, Hicham Benabdelkamel, Anas M. Abdel Rahman

**Affiliations:** 1 Department of Botany and Microbiology, College of Science, King Saud University, Riyadh 11451, Saudi Arabia; 2 Department of Molecular Oncology, King Faisal Specialist Hospital and Research Centre (KFSHRC), Riyadh 11211, Saudi Arabia; 3 Department of Biopharmaceutics and Clinical Pharmacy, School of Pharmacy, The University of Jordan, Amman 11942, Jordan; 4 Proteomics Resource Unit, Obesity Research Center, College of Medicine, King Saud University, Riyadh 11461, Saudi Arabia; 5 Metabolomics Section, Department of Clinical Genomics, Center for Genomics Medicine, King Faisal Specialist Hospital and Research Centre (KFSHRC), Riyadh 11211, Saudi Arabia; 6 Department of Biochemistry and Molecular Medicine, College of Medicine, Al Faisal University, Riyadh 11533, Saudi Arabia; 7 Department of Family Medicine and Polyclinic, King Faisal Specialist Hospital & Research Center, Riyadh 11211, Saudi Arabia; 8 Department of Chemistry, Memorial University of Newfoundland, St. John’s, NL A1C 5S7, Canada

**Keywords:** proteomics, mass spectrometry, bone mineral density (BMD), osteoporosis, osteopenia

## Abstract

Bone mass reduction due to an imbalance in osteogenesis and osteolysis is characterized by low bone mineral density (LBMD) and is clinically classified as osteopenia (ON) or osteoporosis (OP), which is more severe. Multiple biomarkers for diagnosing OP and its progression have been reported; however, most of these lack specificity. This cohort study aimed to investigate sensitive and specific LBMD-associated protein biomarkers in patients diagnosed with ON and OP. A label-free liquid chromatography-mass spectrometry (LC-MS) proteomics approach was used to analyze serum samples. Patients’ proteomics profiles were filtered for potential confounding effects, such as age, sex, chronic diseases, and medication. A distinctive proteomics profile between the control, ON, and OP groups (Q^2^ = 0.7295, R^2^ = 0.9180) was identified, and significant dysregulation in a panel of proteins (*n* = 20) was common among the three groups. A comparison of these proteins showed that the levels of eight proteins were upregulated in ON, compared to those in the control and the OP groups, while the levels of eleven proteins were downregulated in the ON group compared to those in the control group. Interestingly, only one protein, myosin heavy chain 14 (MYH14), showed a linear increase from the control to the ON group, with the highest abundance in the OP group. A significant separation in the proteomics profile between the ON and OP groups (Q^2^ = 0.8760, R^2^ = 0.991) was also noted. Furthermore, a total of twenty-six proteins were found to be dysregulated between the ON and the OP groups, with fourteen upregulated and twelve downregulated proteins in the OP, compared to that in the ON group. Most of the identified dysregulated proteins were immunoglobulins, complement proteins, cytoskeletal proteins, coagulation factors, and various enzymes. Of these identified proteins, the highest area under the curve (AUC) in the receiver operating characteristic (ROC) analysis was related to three proteins (immunoglobulin Lambda constant 1 (IGLC1), RNA binding protein (MEX3B), and fibulin 1 (FBLN1)). Multiple reaction monitoring (MRM), LC-MS, was used to validate some of the identified proteins. A network pathway analysis of the differentially abundant proteins demonstrated dysregulation of inflammatory signaling pathways in the LBMD patients, including the tumor necrosis factor (TNF), toll-like receptor (TL4), and interferon-γ (IFNG) signaling pathways. These results reveal the existence of potentially sensitive protein biomarkers that could be used in further investigations of bone health and OP progression.

## 1. Introduction

The dynamic processes of bone formation maintain bone integrity through bone formation and resorption, which are mediated by bone cells, osteoblasts, and osteoclasts [1]. The activities of these bone cells are naturally balanced, and bone is an active tissue that undergoes constant remodeling [1]. However, defects in osteoblast differentiation and mineralization activities and increased osteoclast activity can result in reduced bone mineral density (BMD), which is clinically graded as osteopenia (ON) or the more severe disease, osteoporosis (OP) [2].

OP is a chronic progressive metabolic bone disease characterized mainly by low bone mineral density (LBMD) and impaired bone microarchitecture, which increases bone fragility and increases the risk of fractures [3]. Several risk factors, including age, gender, estrogen and calcium deficiency, chronic metabolic diseases (such as type 2 diabetes mellitus (T2DM) and hyperthyroidism), reduced physical activity, and medications, such as glucocorticoids have been shown to affect the bone remodeling process and are thus related to the risk of developing OP [1,4,5]. The prevalence of OP is high, particularly in postmenopausal women [6] and in those from developed countries [1]. For example, the OP prevalence rate reaches 48% in older adults within the Saudi population [7].

OP is associated with several complications that can negatively affect a patient’s quality of life. Essential bone fragility fractures associated with OP involve the hip, spine, and wrist [8,9]. Up to 20% of hip fractures result in death within one year following incidence [8,9]. An early LBMD diagnosis can prevent the development of OP and reduce the incidence of bone fragility fractures [10]. The diagnosis of OP is based on measuring BMD using dual-energy X-ray absorptiometry (DXA) [11]. The measured BMD is presented as a *t*-score and calculated as a standard deviation (SD) by considering the mean BMD of peak bone mass in healthy young adults of the same sex [12]. Individuals with a bone mass *t*-score greater than −1.0 are considered to have normal BMD. Patients with a bone mass *t*-score lower than −2.5 are considered to have OP, while those with a *t*-score between −2.5 and −1.0 are considered to have ON [13].

Although OP has been widely studied, the exact molecular mechanisms underlying LBMD have not yet been fully elucidated. A growing line of research has recently focused on identifying circulating proteins and their biological roles in modulating bone metabolism. In this respect, our earlier work revealed significant differences in circulating metabolites between ON and OP patients [14]. Using multi-omics techniques, several BMD-associated proteins have been identified during the profiling of human proteomes in different populations [15,16,17,18]. In addition, several proteomics studies have indicated that proteome-based biomarkers are associated with bone metabolism and OP progression [16,17,18,19,20,21]. However, although the identification of the proteins and metabolic pathways involved in regulating bone metabolism in different populations has increased, precise knowledge of the biological mechanisms underlying LBMD is incomplete, and the existing bone-associated biomarkers are imperfect predictors of bone diseases. Therefore, this study investigated the potential serum proteomics profiles of patients with LBMD by considering proteins associated with confounders and compared them to the profiles of healthy controls. The biological pathways and potential proteins that may contribute to bone loss in patients with OP and ON were also examined. The results of this study provide insights into understanding alterations in proteins associated with LBMD, and they could assist in the discovery of candidate biomarkers for predicting OP progression.

## 2. Results

### 2.1. Clinical Characteristics and Demographics of Study Population

The clinical and demographic characteristics of the study participants are presented in Table 1. Based on their BMD, the participants were divided into the following groups: control (*n* = 22, 31.88%), ON (*n* = 22, 31.88%), and OP (*n* = 25, 36.23%) The study population comprised a higher proportion of females *(n* = 52, 75.36%), and they were mostly postmenopausal (*n* = 51, 98%). There were no significant differences between the body mass indexes (BMIs) of the study groups. However, both the ON and OP groups had significantly lower lumbar *t*-scores, femoral *t*-scores, fasting blood glucose (FBG), triglyceride (TG), and cholesterol levels than the control group (Table 1). Patients in the OP group had a significantly lower lumbar *t*-score than those in the ON group, as shown in Table 1.

### 2.2. Results of Overall Proteomics Analysis and Exclusion of Confounder-Associated Proteins

**Figure 1:**
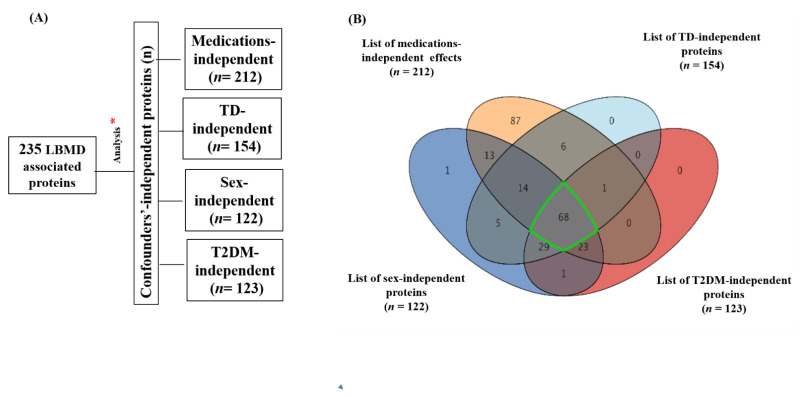
Overall proteomics analysis and exclusion of confounders-associated proteins. (**A**) Determination of confounder-independent proteins from the overall detected proteins. (**B**) Venn diagram demonstrating overlap between confounder-independent proteins (medications, TD, Sex, and T2DM) (*n* = 212, 154, 122, and 123, respectively) using moderate t-test and considering fold change (FC 1.5) and cut-off *p*-value < 0.05. A total of 68 proteins were identified as being significantly associated with LBMD, independent of the effect of confounders. Abbreviations: LBMD: low bone mineral density; TD: thyroid disease; T2DM: type 2 diabetes mellitus. * Two-way ANOVA with FDR-corrected *p*-value (FDRp) cutoff = 0.05.

Initially, 235 proteins were detected, identified, and quantified in the serum samples of the study groups using a proteomics platform. As shown in Table 1, there were more females than males in the cohort; therefore, sex was not matched between the study groups. In addition, some patients with LBMD (ON and OP) had type 2 diabetes mellitus (T2DM) and thyroid disease (TD), and all were taking medication. As these confounding factors might have affected the differential expression of proteins between the study groups, the effect of these confounding factors (T2DM, TD, sex, and medication) on the protein levels was considered in the ultimate profile, as shown in Figure 1.

The Venn diagram analysis shows an overlap between medication-independent (*n* = 212), TD-independent (*n* = 154), sex-independent (*n* = 122), and T2DM-independent (*n* = 123) proteins (Figure 1A) (Supplementary Figure S1). Using a moderate *t*-test and considering a fold change (FC 1.5) and cut-off *p*-value < 0.05, 68 proteins were identified as being significantly associated with LBMD, independent of the effect of confounding factors (Figure 1B) (Supplementary Table S1).

### 2.3. Proteomics Profiles of Control, ON, and OP Groups

**Figure 2:**
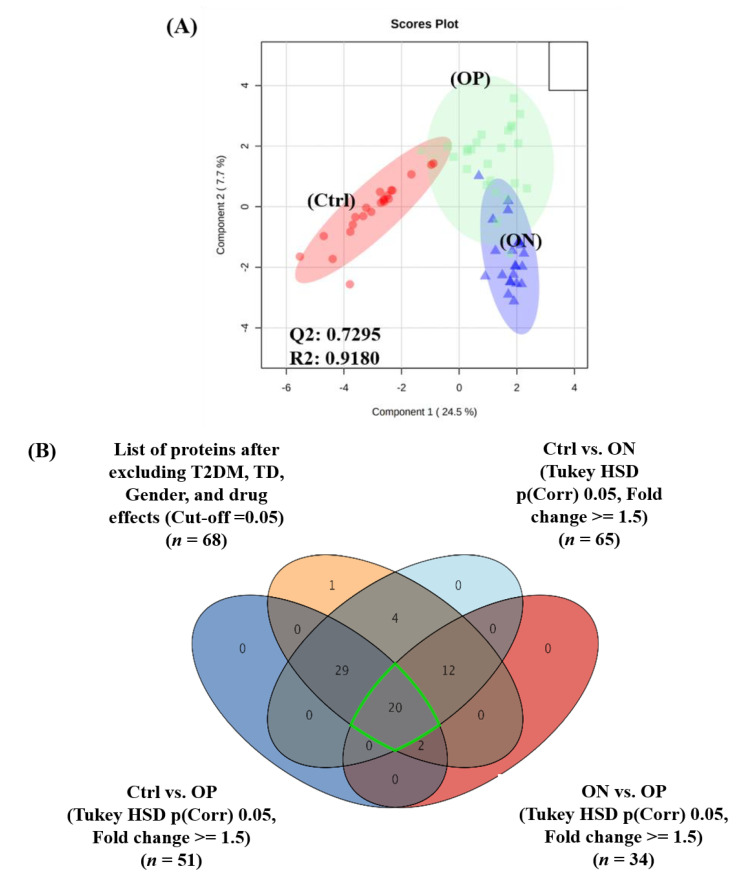

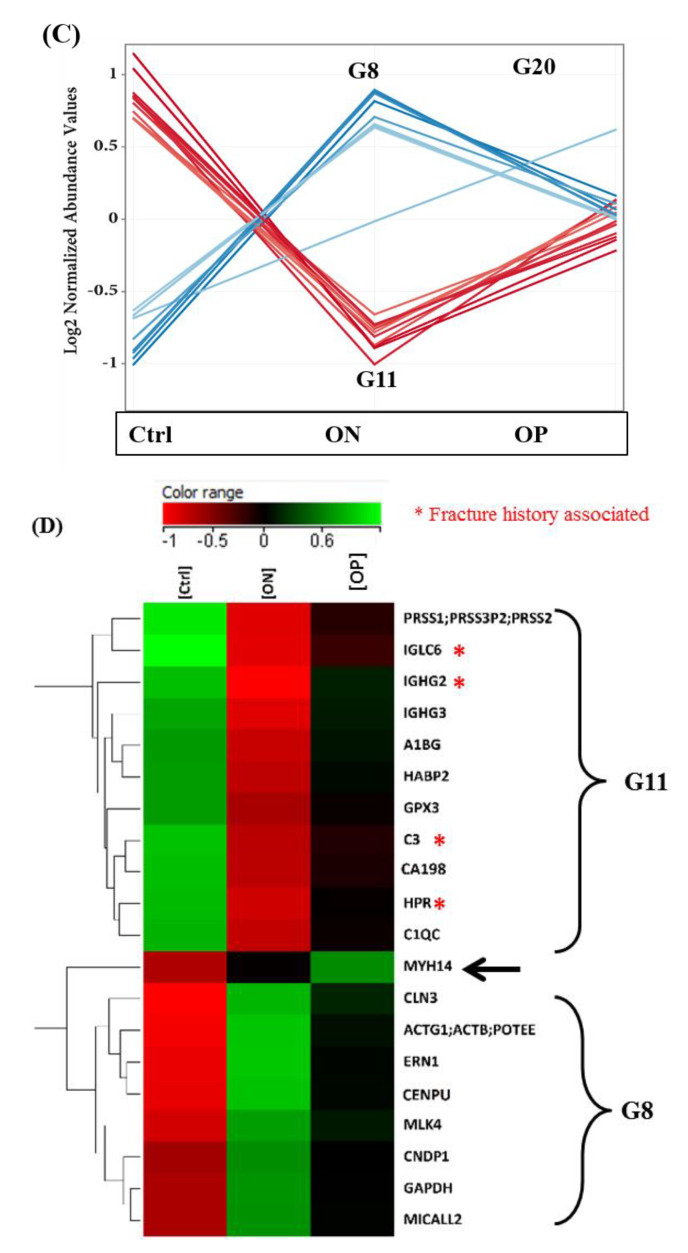
Proteomics profiling between healthy control (Ctrl), osteopenic (ON), and osteoporotic (OP) patients. (**A**) Orthogonal partial least squares-discriminant analysis (OPLS-DA) cross-validation illustrates the significant differences between the three study groups (Ctrl, ON, OP) (Q^2^ = 0.7295, and R^2^ = 0.9180). (**B**) Venn diagram demonstrating the significantly dysregulated proteins between Ctrl vs. ON (*n* = 65), Ctrl vs. OP (*n* = 34), and ON vs. OP (*n* = 51), considering FC of 1.5 and *p*-value of 0.05. Also shown is the identification of common and significant proteins (*n* = 20) between the three study groups. (**C**) Levels of commonly dysregulated proteins (G20) among the three groups, where G8 were upregulated to the highest abundance in the ON group, compared to that in the control group and then downregulated in the OP group, compared to that in the ON group, while G11 was downregulated to the lowest abundance in ON, compared to that in control and then upregulated in OP compared to that in ON. (**D**) Heat map showing the identity and expression levels of the 20 significantly detected proteins among the three study groups and also those associated with fracture history (FH) (highlighted with an asterisk). Green and colors mean down and up-regulation, respectively.

The proteomics pattern associated with the three study groups (control, ON, and OP) was examined using orthogonal partial least squares-discriminant analysis (OPLS-DA) cross-validation (multivariate analysis). As shown in Figure 2A, relative group separation and sample clustering between the control, ON, and OP groups (Q2 = 0.7295, R^2^ = 0.9180) were observed, indicating distinct proteomics differences between these three groups. Comparisons between the control and either the ON or OP groups were conducted using post-hoc Tukey’s analysis (FDR *p*-value < 0.05) and fold change scores (FC 1.5). The results showed that 65 and 51 proteins were significantly dysregulated in the ON and OP groups, respectively, compared to their expression in the control group (Figure 2B). In addition, a comparison between ON and OP groups indicated that 34 proteins were significantly dysregulated between the two groups (Figure 2B). Overlapping of these binary comparison panels and the filtered confounders independent protein panel (*n* = 68) and applying the one-way ANOVA resulted in the identification of 20 significant and common dysregulated proteins among the three groups (control, ON, and OP) (Figure 2B) (Supplementary Table S2). As shown in Figure 2C, the levels of these 20 proteins were either upregulated or downregulated in LBMD (ON and OP groups), compared to that in the control. The levels of eight proteins were upregulated in the ON group, compared to those in the control group but downregulated in the OP group, compared to those in the ON group. In contrast, 11 proteins were downregulated in the ON group, compared to the control but were upregulated in the OP group (Figure 2C). These proteins included immunoglobulins (Igs), complement proteins, enzymes, acute phase proteins (APP), cytoskeletal proteins, binding proteins, and coagulation factors (Figure 2D). The notable proteins identified with an increased abundance in the ON group, compared to that in the control and OP groups (*n* = 8) included CLN3, ACTG1, ERN1, and CENPU. The identified proteins with a decreased abundance in the ON group, compared to that in the control and OP groups (*n* = 11) included IGLC6, IGHG2, IGHG3, C3, HPR, GPX3, and C1QC (Figure 2D).

Interestingly, only one protein (MYH14) showed a linear increase from the control to the ON group, and it was most highly abundant in the OP group (Figure 2C). Fracture history (FH) in patients with LBMD is associated with several serum proteins that have already been identified as biomarkers for bone biology and fracture prediction [22]. Using a moderate t-test and fold-change analysis, this study identified proteins dysregulated by FH. Interestingly, the identity and expression of some FH-associated proteins were highlighted in the heat map (Figure 2D).

### 2.4. Proteomics Profile between ON and OP Groups

**Figure 3:**
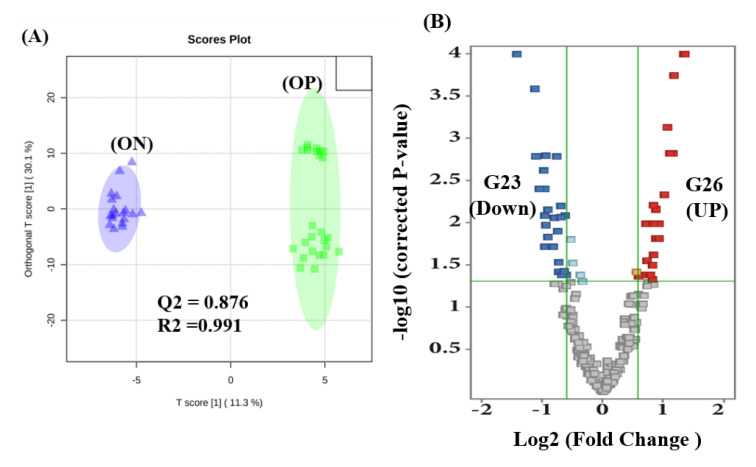

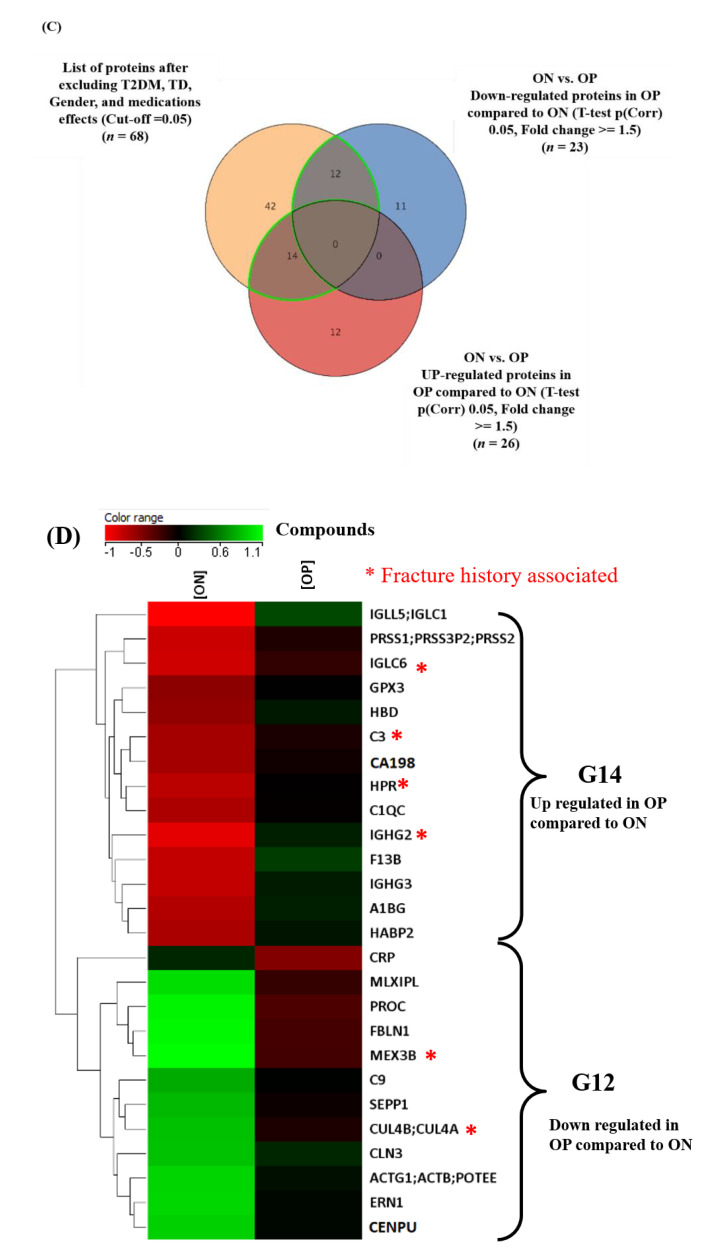
Proteomics profiling between ON and OP patients. (**A**) Orthogonal partial least squares-discriminant analysis (OPLS-DA) score plot showing the relative separation between ON and OP groups (Q^2^ = 0.876, and R^2^ = 0.991) after excluding three outlier values detected using the random forest algorithm. (**B**) Volcano plot analysis of ON versus OP showing significantly dysregulated proteins (false discovery rate (FDR)-corrected *p*-value < 0.05, and fold change (FC) > 1.5 or < 0.67). A total of (G49) proteins were found to be dysregulated (26 up-regulated and 23 down-regulated) in OP patients, compared to those in ON patients. (**C**) Venn diagram illustrating an overlap between the confounder’s independent proteins (*n* = 68) and the dysregulated proteins between the ON and OP groups (G49). A total of 26 proteins were significantly dysregulated (14 up-regulated and 12 down-regulated) in OP, compared to those in ON patients. (**D**) Heat map showing the expression and the identity of the dysregulated proteins between the ON and OP groups along with fracture history (FH)-associated proteins (highlighted with an asterisk). Green and colors mean down and up-regulation, respectively.

Although participants in the ON and OP groups belonged to the LBMD group, and they thus had approximately similar physiological bone changes, the proteomics profile associated with each separate condition was examined. Interestingly, the OPLS-DA analysis presented clear group separation and sample clustering between the ON and OP groups (Q^2^ = 0.876, R^2^ = 0.991) (Figure 3A). A volcano blot analysis conducted between the ON and OP groups using a moderate t-test (*p*-value < 0.05) and fold change (FC cutoff of 1.5) indicated that 26 and 23 proteins were upregulated and downregulated, respectively, in the OP group, compared to that in the ON group (Figure 3B). An overlap using a Venn diagram analysis of the filtered confounders’ independent proteins (*n* = 68), and the dysregulated proteins between the ON and OP groups (26 upregulated and 23 downregulated) revealed 26 dysregulated proteins (Figure 3C) (Supplementary Table S3); of these 26 proteins, 14 were upregulated and 12 were downregulated in the OP group, compared to that in the ON group, as shown in Figure 3C. The identification and clustering of dysregulated proteins in the ON and OP groups and FH-associated proteins are highlighted within the heat map (Figure 3D).

### 2.5. Evaluation of Biomarkers between ON and OP

**Figure 4:**
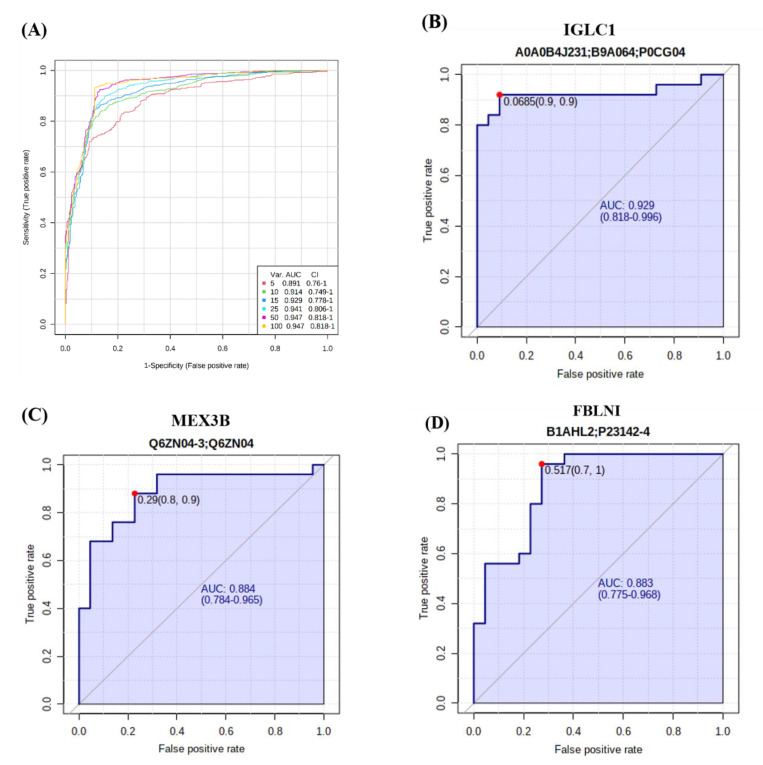
Results of biomarker evaluation in ON and OP. (**A**) Exploratory ROC curve generated by the OPLS-DA model; AUC values were calculated by mathematical integration of the combination of 5, 10, 15, 25, 50, and 100 proteins. (**B**–**D**) Three proteins with the highest AUC: (**B**) IGLC1, AUC = 0.929; (**C**) MEX3B, AUC = 0.884; and (**D**) FBLNI, AUC = 0.883.

A multivariate exploratory ROC analysis based on the identified common and significantly dysregulated proteins between the ON and OP (*n* = 26) groups was performed using OPLS-DA as a classification and feature ranking method. The AUC of the exploratory ROC curve for the top ten variants (proteins) was 0.914 (Figure 4A). Interestingly, the AUCs of three proteins were the highest: IGLC1 (AUC of 0.929, Figure 4B), MEX3B (AUC of 0.884, Figure 4C), and FBLN1 (AUC of 0.883, Figure 4D).

**Figure 5:**
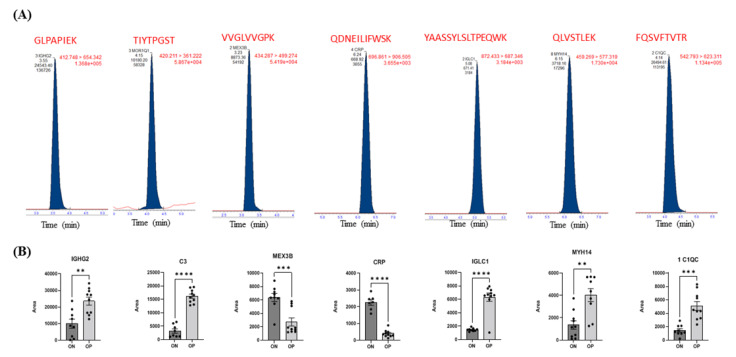
Validation of the expression of selected proteins in the ON and OP groups using multiple reaction monitoring (MRM). (**A**) Representative MRM chromatograms for protein signature peptides selected from the Skyline Software and confirmed using PeptideAtlas. (**B**) Scatter plots with bar-graph for the expressions of selected proteins. The differences between the study groups were evaluated using an unpaired *t*-test with significance set at *p*-value <0.01 (denoted by **), <0.001 (denoted by ***), and <0.0001(denoted by ****).

Seven different proteins (IGHG2, C3, MEX3B, CRP, IGLC1, MYH14, and C1QC) were selected from the proteomics profiles of the ON and OP groups to conduct a targeted protein analysis and validation of protein expression using multiple reaction monitoring (MRM), which is a highly specific and sensitive MS technique used to quantify proteins. These selected proteins were among the significantly dysregulated proteins in the ON and OP groups. Based on the proteomics profile shown in the heat map (Figure 3D), IGHG2, C3, IGLC1, and C1QC were upregulated in OP patients, compared to those in ON patients, and MEX3B and CRP levels were downregulated in OP patients, compared to those in ON patients (Figure 3D). Interestingly, the MRM results confirmed these findings (Figure 5A, B).

### 2.6. Network Module Analysis and Biological Pathways Related to ON and OP

**Figure 6:**
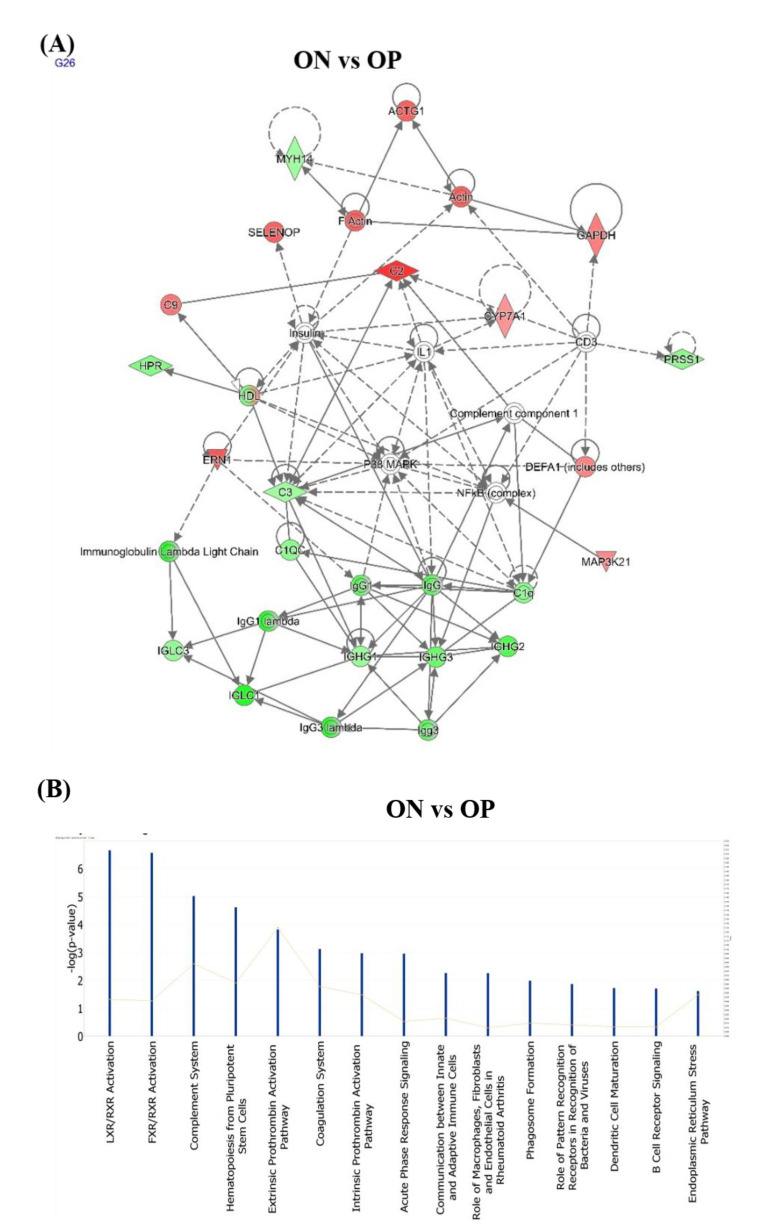
Network analysis and biological pathways related to the significantly identified proteins in the study population. (**A**) Network pathway analysis of the significantly dysregulated proteins identified in the ON group, compared to those in the OP group revealed that they were related to the developmental disorder, hereditary disorder, and metabolic disease. The analysis also showed the involvement of the TNF and IFNG signaling pathways. (**B**) The top canonical pathways related to the significantly dysregulated proteins identified in the ON group, compared to those in the OP group.

Ingenuity pathway analysis (IPA) software was used to perform a network analysis to investigate the potential pathways associated with significantly identified serum proteins related to ON and OP development. The highest-scoring network pathways identified in the ON and OP groups were the humoral immune response, inflammatory response, and developmental disorder (Score 52) (Figure 6A). Moreover, the top four potentially significantly enriched pathways in the ON versus OP group included the following: LXR/RXR activation, *p* = 7.62 × 10^−6^ (with an overlap of 3.4%, 4/117), FXR/RXR activation *p* = 8.70 × 10^−6^ (with an overlap of 3.3%, 4/121), complement system *p* = 5.71 × 10^−8^ (with an overlap of 11.4%, 4/35), and hematopoiesis from pluripotent stem cells *p* = 2.29 × 10^−7^ (with an overlap of 8.2%, 4/49) (Figure 6B).

## 3. Discussion

The label-free proteomics approach employed in this study revealed a pattern of serum proteins that were associated with ON and OP patients and not with the healthy controls. The findings of this study will enable the screening of possible protein biomarkers related to either risk prediction or the progression of ON and OP.

### 3.1. Proteomics Profiles of Control, ON, and OP Groups

Chronic metabolic diseases, such as T2DM and TD, are commonly associated with LBMD. In addition, medication consumed by patients influences bone metabolism [23,24,25]. Therefore, in addition to sex, the impact of these confounders was excluded from the proteomics profiles of the LBMD groups (ON and OP groups). The results showed 68 proteins that were independent of the determined confounders, and they were used to study the protein expressions associated with ON and OP.

Our results also showed an evident group separation of significantly and differentially expressed proteins between the control, ON, and OP groups. However, 20 proteins (*n* = 20) were significantly dysregulated in all three groups; of these, only one protein (MYH14) showed a linear increase from the control to the ON group, with the highest abundance occurring in the OP group. These findings agree with those of other omics studies that have identified MYH14 as a significant protein associated with LBMD [15]. MYH14 is a member of the myosin superfamily, and it belongs to the unconventional non-muscle myosin II complex, which plays an important role in cytoskeletal organization, osteoclast podosome formation, and bone resorption [26,27]. Moreover, MYH14 is involved in the phosphorylation of MYL9, which increases the formation of mature osteoclasts [15,28]. MYH14 is also associated with osteoclast activity and bone resorption [15,28]. Although the mechanisms involved in osteoclast differentiation have not been entirely examined, the linear increase in the levels of this protein among the control, ON, and OP groups may indicate an upregulation of osteoclast activity at multiple levels during the progression from ON to OP.

### 3.2. Fracture History-Associated Proteins in Control, ON, and OP Groups

BMD is an important determinant of fracture risk [29], and evidence indicates that FH in patients with LBMD is associated with the existence of several serum proteins that have been identified as biomarkers for bone biology and fracture risk prediction [22]. In this study, six proteins were found to be connected to FH in patients with LBMD, and they could thus be used in fracture risk estimation. Of these FH-associated proteins, four (IGHG2, IGLC6, C3, and HPR) were dysregulated in the control, ON, and OP groups. This observation supports the connection between potential protein biomarkers associated with fracture risk and the progress of bone loss in ON and OP patients.

### 3.3. Comparison of Proteomics Profiles between ON and OP Groups: Role of the Immune System

Of the 26 proteins that were dysregulated in the ON and OP groups, 14 and 12 were upregulated and downregulated, respectively, in the OP group, compared to that in the ON group. Most of the identified dysregulated proteins were related to the immune system (Igs and complement). New emerging studies have recognized the complex interactions between bones and the immune system, which has led to the discovery of an interdisciplinary field known as osteoimmunology [30,31]. Our proteomics analysis revealed a significant dysregulation in Ig levels (including heavy (IGHGs) and light (IGLCs) chains) in the ON and OP groups, compared to those in the control group.

Previous studies have emphasized the role of B cells in controlling bone integrity [32]. Mature B cells not only synthesize Ig, but they also secrete signaling molecules (such as receptor activators of nuclear factor-kappa κB ligand (RANKL) and osteoprotegerin (OPG) that regulate bone homeostasis through their actions on osteoblasts and osteoclasts [33,34]. The upregulation of Igs in OP patients, compared to that in ON patients indicates dysregulation of the immune system in these patients. Our study also showed that patients with LBMD (OP and ON) had upregulated levels of proinflammatory cytokines, including tumor necrosis factor-α (TNF-α) and interleukin-6 (IL-6), compared to the controls.

The results of this study also showed significant dysregulation in complement proteins, including C3 and the complement C1q C chain (C1QC) (upregulated in OP compared to that in ON) and C9 (downregulated in OP compared to that in ON). Furthermore, the results of this study showed that complement system was one of the enriched canonical pathways detected by IPA in LBMD patients. In addition to their role in immunity, complement proteins are important signaling molecules and regulators of bone growth, homeostasis, and cartilage-to-bone transition that critically influences crosstalk between osteoblasts and osteoclasts [35]. C3 is a central molecule in the complement cascade, and it has a known association with proinflammatory activity [36].

In contrast, C1QC is a subunit of the C1 complex [37]. An increase in these complement components in the OP group indicates inflammation and an increase in osteoclast activity. However, complement protein C9, a component of the membrane attack complex expressed during bone formation and ossification (osteogenesis) [38] was found to be decreased in the OP patients, compared to that in ON patients. These findings indicate that the progression of ON to OP involves an inflammatory response associated with an increase in the activity of osteoclasts and a decrease in osteoblast activity, and thus low bone formation.)

### 3.4. Common and Significant Proteins Biomarkers and Pathways in ON and OP

The AUC under the curve was the highest for three proteins (Immunoglobulin Lambda Constant 1 (IGLC1), extracellular matrix protein Fibulin 1 (FBLN1), and Mex-3 RNA Binding Family Member B (MEX3B)) in the ROC analysis. IGLC1 was upregulated, while the levels of both MEX3B and FBLN1 were downregulated in the OP group, compared to those in the ON group. FBLN1 is involved in cytoskeletal physiology and extracellular matrix (ECM)-receptor interactions. These findings are consistent with those of a previous bioinformatic analysis that determined the downregulation of cytoskeletal proteins in OP [17]. In addition, an animal study using an FBLN1 deficient mice model indicated the role of FBLN1 in bone formation and ossification [39]. MEX3B is known to be involved in post-transcriptional regulatory mechanisms, but its role in bone turnover remains unclear. Our results show that this protein is associated with FH, which is a strong predictor of future osteoporotic fractures.

Liver X receptor (LXR) and retinoid X receptor (RXR) signaling are crucial modulators of bone remodeling [40]. Activation of the LXR pathway inhibits RANKL-mediated osteoclast differentiation, whereas retinoid X receptors (RXRs) are key elements in the transcriptional program of differentiating osteoclasts [41]. The role of LXR/RXR activation ligands as therapeutic agents in bone diseases, such as osteoporosis, is emerging [40]. In addition, in vitro and in vivo studies have demonstrated the role of farnesoid X receptor (FXR); this nuclear receptor functions as a bile acid sensor that regulates bone metabolism through its effect on bone remodeling pathways [42]. In this respect, the IPA revealed that activation of the LXR/RXR and FXR/RXR pathways was the most significantly enriched canonical pathway related to proteins identified in ON, compared to that in OP.

## 4. Materials and Methods

### 4.1. Study Population and Recruitment

For this exploratory cohort study, 69 participants aged 50 years, and comprising people of both sexes, were recruited from the osteoporosis clinic at King Faisal Specialist Hospital and Research Center (KFSHRC), Riyadh, Saudi Arabia. Lumbar and femoral *t*-scores were measured using dual-energy X-ray absorptiometry (DXA) scan. According to the BMD *t*-score, the participants were divided into three groups: control (*t*-score > −1.0, *n* = 22), osteoporosis (OP) (*t*-score < −2.5, *n* = 25), and osteopenia (ON) (*t*-score = −2.5 to −1, *n* = 22). Both the ON and OP groups were categorized as the LBMD group. Participants with cancer and chronic diseases, such as infectious arthritis and lung, cardiovascular, liver, and renal diseases, were excluded. In addition, patients taking medication, such as glucocorticoids or hormonal replacements (estrogen and androgen therapy), were excluded. The demographic and clinical data of participants were collected using an approved questionnaire during recruitment.

### 4.2. Ethical Approval

All procedures used in this study, including those involving human participants, were conducted in accordance with the ethical standards stipulated in the guidelines of the Declaration of Helsinki and the Universal International Conference on Harmonization-Good Clinical Practice (ICH-GCP) guidelines. This study was reviewed and approved by the Institutional Review Board (IRB) of King Faisal Specialist Hospital and Research Center (KFSHRC) (RAC #2180 003), Riyadh, Saudi Arabia. All participants signed consent forms prior to being enrolled in the study.

### 4.3. Proteomics Analysis

#### 4.3.1. Protein Digestion and Sample Preparation

Serum samples from the study group were initially processed using immunoaffinity spin columns (Sigma-Aldrich, St. Louis, MO, USA) to remove highly abundant proteins, human serum albumin (HSA), and IgG. The depleted proteins were precipitated overnight with acetone at −20 °C, and the pellets were dissolved in 200 μL ammonium bicarbonate (50 mM). Disulfide bonds were reduced by incubation in the presence of dithiothreitol (DTT) (Sigma, St. Louis, MO, USA) (10 mM) at 56 °C for 1 h, and then the proteins were alkylated with iodoacetamide (IAA) (Sigma, St. Louis, MO, USA) (20 mM) at room temperature in the dark for another hour. Protein samples were digested using trypsin (Promega, Madison, WI, USA) at a trypsin-to-protein ratio of 1:50 and incubated at 37 °C overnight. Protein digests were resuspended in 20 μL of 0.1% formic acid prior to conducting the liquid chromatography-mass spectrometry LC-MS/MS analysis.

#### 4.3.2. Nano LC-MS/MS Analysis

Tryptic peptide separation was performed on an Ultimate 3000 nano-LC system coupled with a source of nanoelectrospray ionization (ESI). The ionized peptides were separated using a Q Exactive HF mass spectrometer (Thermo Fisher Scientific, USA). Protein digests (1 μg) were injected into a trapping column (PepMap C18, 100 Å, 100 μm × 2 cm, 5 μm) and an analytical column (PepMap C18, 100 Å, 75 μm × 50 cm, 2 μm). The tryptic peptides were then separated using solvent A (water with 0.1% formic acid) and solvent B (80% acetonitrile with 0.1% FA) at a flow rate of 250 nL/min, using gradient elution (2% to 8% buffer B for 3 min, 8 to 20% buffer B for 53 min, 20 to 40% buffer B for 35 min, and then 40 to 90% buffer B for 4 min). The full scan was conducted between 300–1650 *m*/*z* at a resolution of 60,000 at 200 *m*/*z*, and the automatic gain control target for the full scan was set to 3e6. The MS/MS scan was operated in top 20 mode using the following settings: resolution 15,000 at 200 *m*/*z*; automatic gain control target of 1 × 10^5^; maximum injection time of 19 mS; normalized collision energy of 28%; isolation window of 1.4 Th; charge state exclusion: unassigned, 1, >6; and dynamic exclusion of 30 s.

### 4.4. LC-MS/MS—Multiple Reaction Monitoring (MRM)

Seven different proteins were selected from the proteomics profile of the ON and OP groups to validate their expressions using multiple reaction monitoring (MRM), which is a highly specific and sensitive MS technique used to quantify proteins. Using the criteria described previously [5,43], at least one signature peptide per protein was identified using the Skyline Software v21. The suggested MRM transitions were exported to a triple-quadrupole-tandem mass spectrometer (XEVO TQmicro, Waters Corporation, Boston, MA, USA). A control-extracted sample was used to evaluate transitions and to optimize the collision energy and column retention time. The patients’ samples were tryptic digested and solid-phase extracted using the standard protocol reported by Galal et al., 2021 [44]. The extracted tryptic peptides were separated by chromatography using an Acquity ultra-performance liquid chromatography (UPLC; AQUITY BEH C18, 1.7 μm, 2.1 mm × 100 mm column; at 25 °C) at a mobile phase flow rate of 0.3 mL/min over a total run time of 12 min. The gradient profile for solvent A (0.1% formic acid in H_2_O) was 90% for 1 min, followed by a linear gradient to 10% over 10 min, which was then held at 10% for 1 min before being returned to 90% in 2 min. For positive-mode mass spectrometric resolution, the eluted peptides were subjected to electrospray ionization (ESI). The following settings were used: source desolvation temperature of 450 °C, desolvation gas flow set at 700 L/h, cone gas flow of 50 L/h, MS capillary source voltage of 1.}98 KV, and cone source of 47 V. The total run time for each sample was 12 min at a mobile phase flow rate of 0.3 mL/min following the gradient table. The samples were stored in an autosampler at 4 °C, with an injection volume of 5 μL. During the run, frequent intermediate washing steps were performed to minimize the sample carryover.

### 4.5. Database Search

Raw data were searched in the human protein database based on the species of samples and using MaxQuant (v1.6.2.6, http://www.maxquant.org accessed on 8 June 2022). Trypsin was used as the cleavage enzyme, and up to two maximum missed cleavages without any modifications were allowed. The precursor ion mass tolerance was set to 10 ppm, and the MS/MS tolerance was 0.6 Da. Only highly confidently identified peptides were selected for downstream protein identification analysis using Universal Protein Resource (Uniprot) database (https://www.uniprot.org/, accessed on 1 June 2022).

### 4.6. Statistical Analysis

The raw data were normalized to the total sample median, log-transformed, and Pareto-scaled to ensure a Gaussian distribution of the signals. Univariate analysis using a volcano plot was performed for each binary comparison to identify significantly differentially expressed proteins based on a fold-change (FC) criterion greater than 1.5 or less than 0.67, with a false discovery rate (FDR) adjusted p-value of less than 0.05. The *x*-axis and *y*-axis on the volcano plot represented the FC between the two comparison groups and the *p*-value, respectively. Multivariate analysis and orthogonal partial least squares-discriminant analysis (OPLS-DA) were performed to identify any clustering or separation between the compared datasets.

To analyze the statistics between the three groups, an analysis of variance (ANOVA) using post-hoc Tukey’s analysis method with multiplicity-adjusted *p*-values for each comparison was used, as this was considered to be the most appropriate method for reducing the probability of type 1 errors. As seen in our cohorts, this method supports the testing of pairwise differences that occur because of unequal group sizes between the experimental and control groups. The data were homogenous and normally distributed, and two assumptions were made using ANOVA. Pearson’s similarity test, hierarchical clustering combined with heat maps, and Venn diagram analysis (including a two-way ANOVA) were performed between the study groups using multiple professional profiler (MPP) software (Agilent In., Santa Clara, CA, USA).

The list of significantly identified proteins was entered into the pathway analysis module to define the biological significance of the identified proteins in the LBMD groups (ON and OP) and their relation to the dysregulation of metabolic pathways. Ingenuity pathway analysis (IPA) software was used to identify potential pathways associated with the significantly identified proteins related to LBMD development, ON, and OP. The generated network maps provide a means of visualizing protein–protein interactions between the identified proteins, both directly and indirectly. Potential biomarkers were evaluated by conducting the receiver operating characteristic ROC curve analysis using MetaboAnalyst Software V5 (Montreal, QC, Canada).

## 5. Conclusions

This study used a label-free proteomics approach and employed LC-MS to analyze the proteomics profiles of patients with different stages of LBMD (ON and its more severe counterpart, OP), compared to those of the healthy controls. A distinctive panel of dysregulated proteins was identified, and they mostly comprised immunoglobulins, complement proteins, cytoskeletal proteins, coagulation factors, and various enzymes. Interestingly, the AUCs in the ROC analysis were the highest for three proteins (IGLC1, MEX3B, and FBLN1), which indicates their significant association with OP. A network analysis of differentially abundant proteins indicated that inflammatory signaling pathways were dysregulated in the ON and OP groups. The results of this study provide insights into the pathways and proteins involved in bone metabolism, and they can be used as a guide to enable the optimum monitoring and treatment of bone diseases.

## Figures and Tables

**Table 1:** Clinical characteristics and demographics of the study population (n = 69).

	Ctrl		ON		OP	
Total n (%)	22 (31.88)		22 (31.88)		25 (36.23)	
Parameters	Mean	SEM	Mean	SEM	Mean	SEM
Age (years)	54.82	1.03	64.64 ^§^	1.72	66.16 ^§^	1.78
Gender (F/M)	(13/9)	-	(15/7)	-	(24/1)	-
Menopause * (Yes/No)	(13/0)	-	(14/1)	-	(24/0)	-
Weight (kg)	85.13	3.63	74.21	3.88	69.23 ^§^	2.86
Height (cm)	162.22	0.02	157.11	0.021	150.68 ^§ ‡^	0.01
BMI (kg/m^2^)	32.21	1.1	30.38	1.84	30.70	1.4
Lumbar t score	0.29	0.24	−1.25 ^§ ‡^	0.21	−2.62 ^§^	0.12
Femoral t score	0.34	0.29	−1.51 ^§ ‡^	0.14	−1.93 ^§^	0.13
FBG (mmol/L)	10.2	1.16	6.08 ^§^	0.39	5.87 ^§^	0.41
HDL (mmol/L)	1.00	0.80	1.47 ^§^	0.12	1.42 ^§^	0.09
TG (mmol/L)	1.85	0.15	1.23 ^§^	0.11	1.127 ^§^	0.08
Cholesterol (mmol/L)	5.51	0.23	4.47 ^§^	0.19	4.27 ^§^	0.29
Calcium (mmol/L)	2.24	0.026	2.37 ^§^	0.025	2.33 ^§^	0.02
Albumin (g/L)	37.65	1.14	41.98 ^§^	2.0	42.75 ^§^	0.86
Vitamin D 25 hydroxy (nmol/L)	68.32	7.39	77.64	3.3	86.57	6.05

Abbreviations: ON: osteopenic, OP: osteoporotic, BMI: body mass index, FBG; fasting blood glucose, LDL; low-density lipoprotein, HDL; high-density lipoprotein, TG; triglycerides. Data are presented as mean ± standard error of the mean (SEM); * menopause status in females; ^§^ *p*-value < 0.05 vs. control group; ^‡^ *p*-value < 0.05 vs. OP group.

## Supplementary Materials

The following supporting information can be downloaded at: https://www.mdpi.com/article/10.3390/ijms231710200/s1
