# Supplementary material for: Proteomics Profiling of Osteoporosis and Osteopenia Patients and Associated Network Analysis
Source: Int J Mol Sci. 2022 Sep 5;23(17):10200. doi: 10.3390/ijms231710200 (PMC9456664; doi:10.3390/ijms231710200)
Supplement: Supplementary file 1 [file ijms-23-10200-s001.zip › modified Supplemntary Figures_27.8.2022.pptx]

## Slide 1
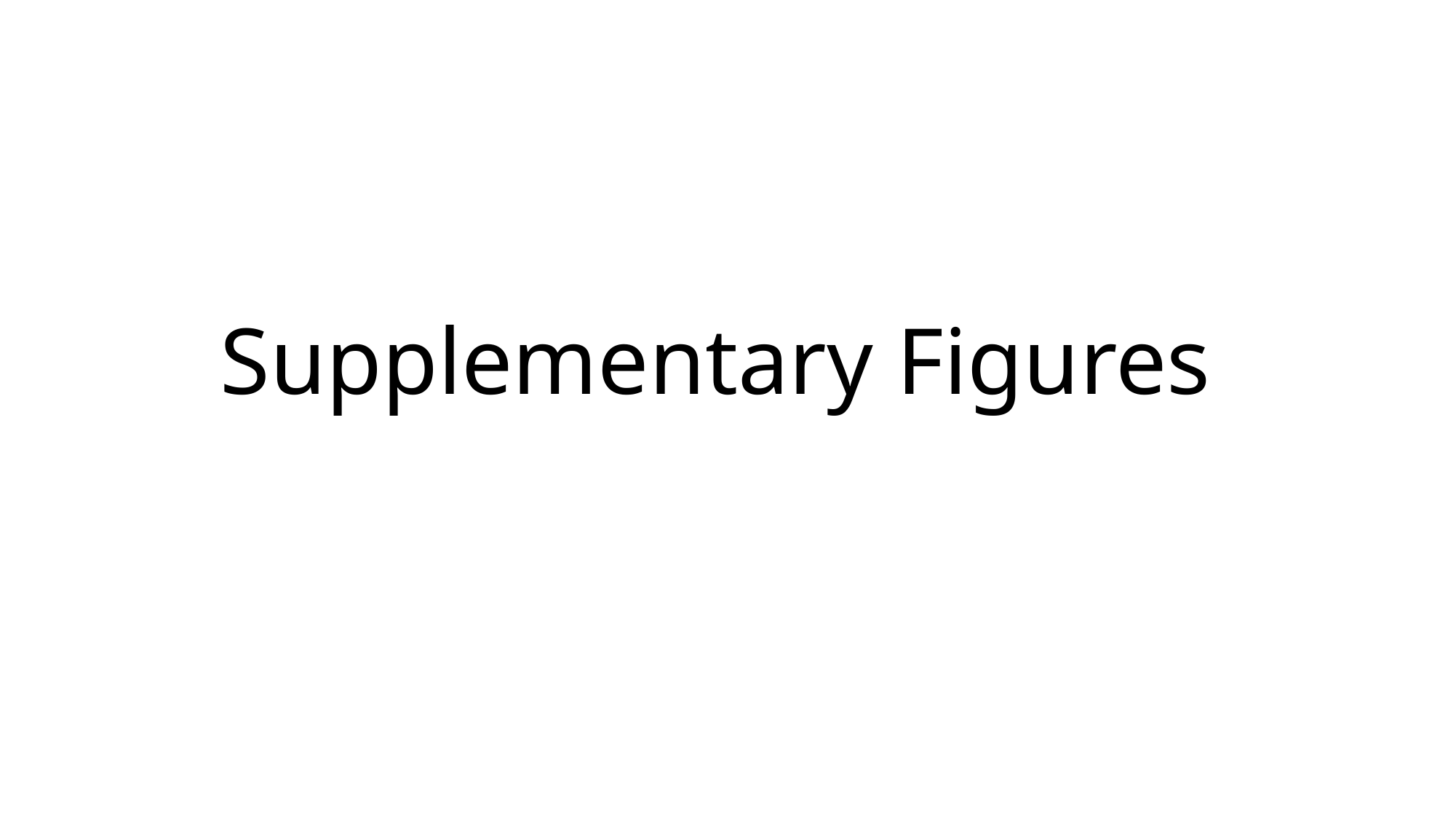

# Supplementary Figures

## Slide 2
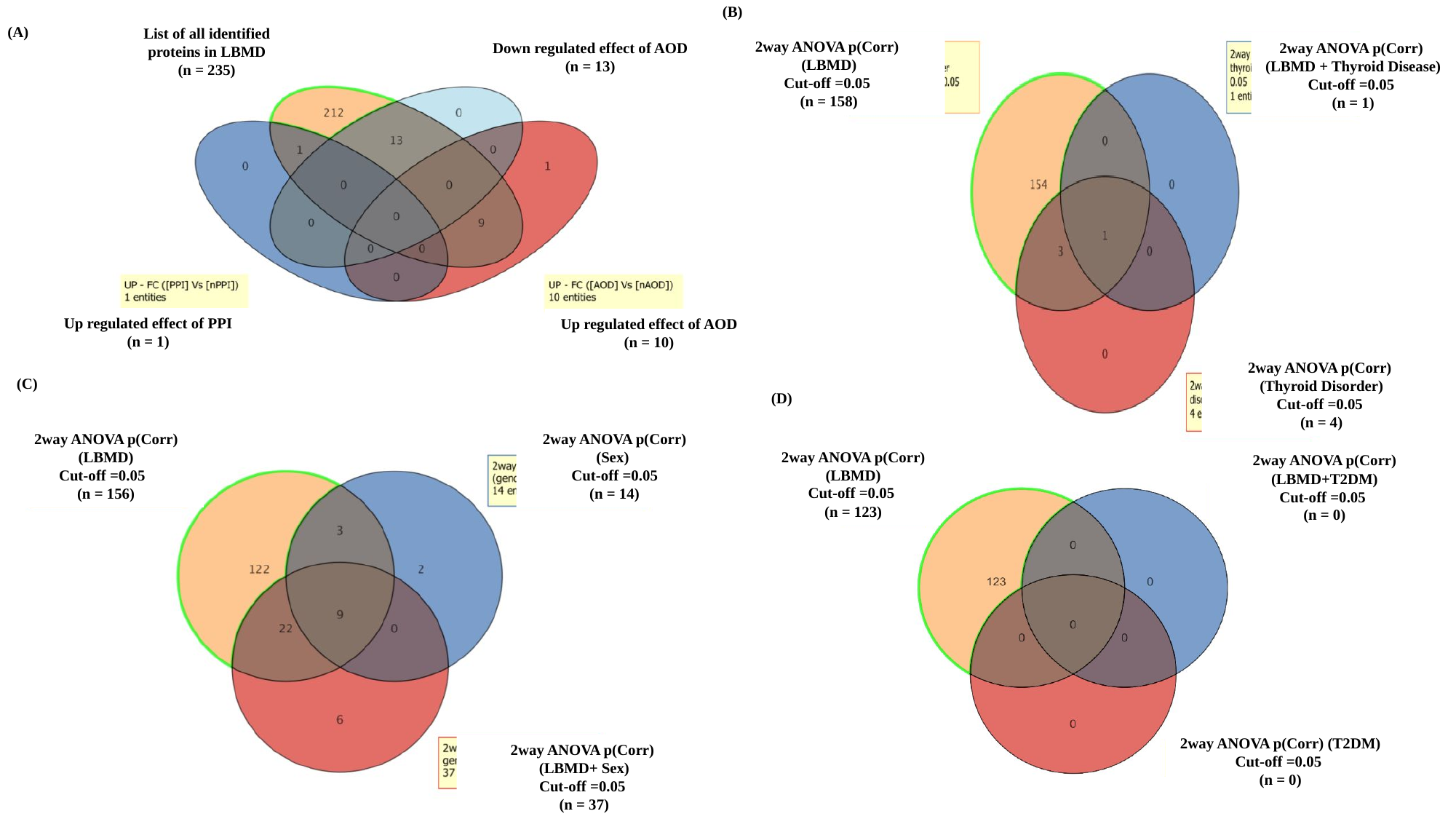

(B)
Down regulated effect of AOD
(n = 13)
List of all identified proteins in LBMD
(n = 235)
Up regulated effect of PPI
(n = 1)
Up regulated effect of AOD
(n = 10)
(A)
2way ANOVA p(Corr)
(LBMD)
Cut-off =0.05
(n = 158)
2way ANOVA p(Corr)
(LBMD + Thyroid Disease)
Cut-off =0.05
(n = 1)
2way ANOVA p(Corr)
(Thyroid Disorder)
Cut-off =0.05
(n = 4)
(C)
(D)
2way ANOVA p(Corr) (LBMD)
Cut-off =0.05
(n = 156)
2way ANOVA p(Corr) (Sex)
Cut-off =0.05
(n = 14)
2way ANOVA p(Corr)
(LBMD+ Sex)
Cut-off =0.05
(n = 37)
2way ANOVA p(Corr) (LBMD)
Cut-off =0.05
(n = 123)
2way ANOVA p(Corr) (LBMD+T2DM)
Cut-off =0.05
(n = 0)
2way ANOVA p(Corr) (T2DM)
Cut-off =0.05
(n = 0)

## Slide 3
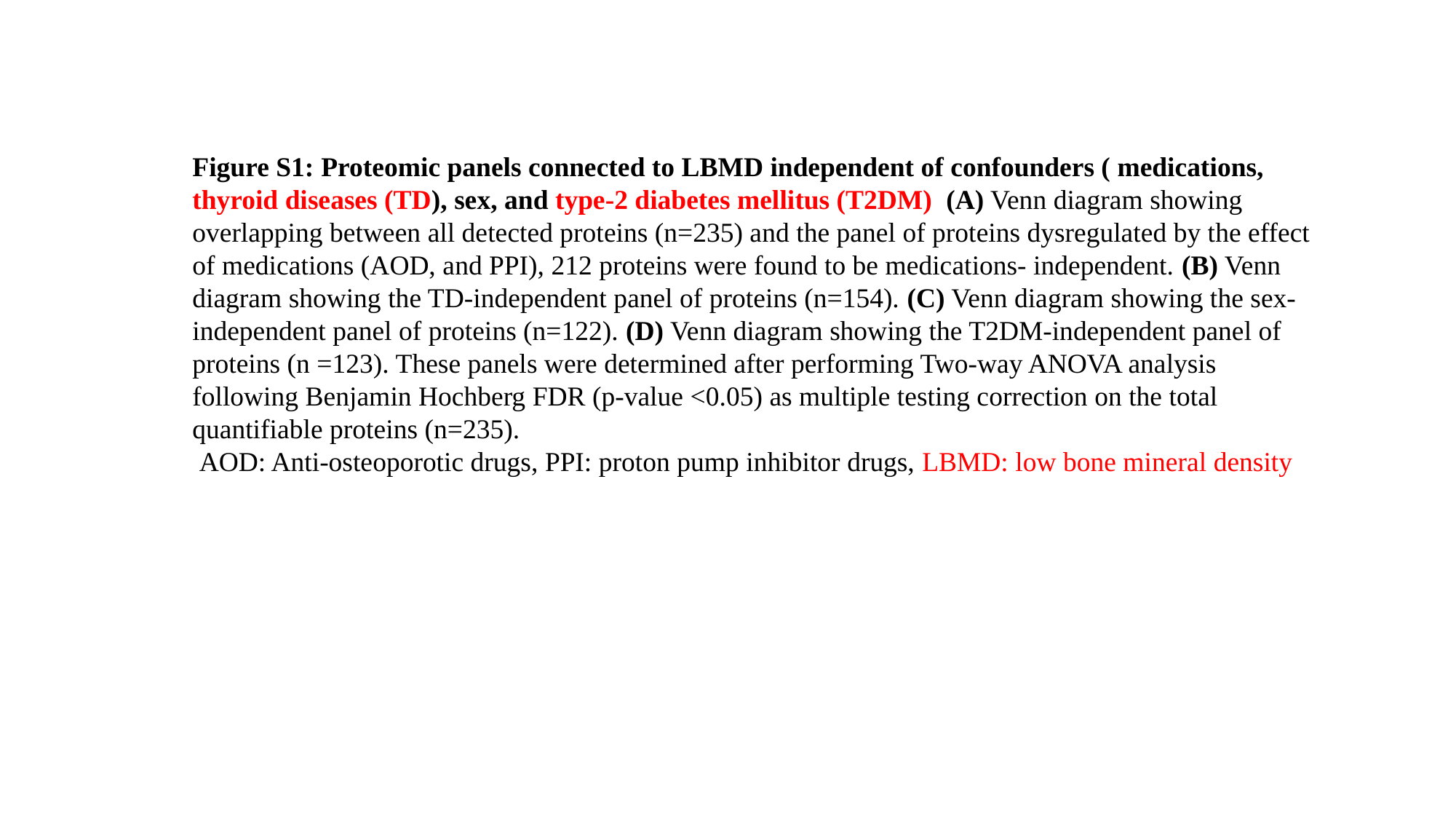

Figure S1: Proteomic panels connected to LBMD independent of confounders ( medications, thyroid diseases (TD), sex, and type-2 diabetes mellitus (T2DM) (A) Venn diagram showing overlapping between all detected proteins (n=235) and the panel of proteins dysregulated by the effect of medications (AOD, and PPI), 212 proteins were found to be medications- independent. (B) Venn diagram showing the TD-independent panel of proteins (n=154). (C) Venn diagram showing the sex-independent panel of proteins (n=122). (D) Venn diagram showing the T2DM-independent panel of proteins (n =123). These panels were determined after performing Two-way ANOVA analysis following Benjamin Hochberg FDR (p-value <0.05) as multiple testing correction on the total quantifiable proteins (n=235).
 AOD: Anti-osteoporotic drugs, PPI: proton pump inhibitor drugs, LBMD: low bone mineral density
