# Supplementary material for: Proteomics Profiling of Osteoporosis and Osteopenia Patients and Associated Network Analysis
Source: Int J Mol Sci. 2022 Sep 5;23(17):10200. doi: 10.3390/ijms231710200 (PMC9456664; doi:10.3390/ijms231710200)
Supplement: Supplementary file 1 [file ijms-23-10200-s001.zip › Supplementary Table S1_29.8.2022.pdf]

Supplementary Table S1: The significant proteins (G65) associated with LBMD and independent of the effect of confounding factors.

Number	Uniprot ID	Protein Name	Accession Number	Fold Change Ctrl/LBMD
1.	A0A075B7B8	Immunoglobulin heavy variable 3	A0A075B7B8	2.30
2.	CBPB2	Carboxypeptidase B2	Q96IY4	-1.96
3.	NCHL1	Neural cell adhesion molecule L1-like protein	O00533	-1.88
4.	GPX3	Glutathione peroxidase 3	P22352	1.85
5.	FA5	Activated protein C cofactor	P12259	-1.90
6.	IGLC1	Immunoglobulin lambda constant 1	P0CG04	2.18
7.	MLXPL	MLX-interacting protein-like	Q9NP71	-1.98
8.	HV102	Immunoglobulin heavy variable 1-2	P23083	1.79
9.	CFH	Complement factor H	P08603	-2.11
10.	TRY1	Trypsin-1	P07477	2.85
11.	SEPP1	Selenoprotein P (SeP)	P49908	-2.06
12.	CLN3	Battenin	Q13286	-2.76
13.	IGHG3	Immunoglobulin heavy constant gamma 3	P01860	2.07
14.	IGHG2	Immunoglobulin heavy constant gamma 2	P01859	2.36
15.	MYH14	Myosin-14 (	Q7Z406	-2.14
16.	FBLN1	Fibulin-1 (FIBL-1)	P23142	-2.05
17.	IGJ	Immunoglobulin J chain	P01591	2.03
18.	PROC	Anticoagulant protein C	P04070	-1.93
19.	TRFL	Lactotransferrin	P02788	-2.08
20.	G3P	Glyceraldehyde-3-phosphate dehydrogenase	P04406	-2.02
21.	F2Z2G5	WAP four-disulfide core domain protein 3	Q8IUB2	2.08
22.	KLKB1	Plasma kallikrein (Fragment)	P03952	2.06
23.	J3KRP0	Beta-Ala-His dipeptidase	Q96KN2	-1.98

24.	M0R1Q1	Complement C3 (Fragment)	P01024	2.35
25.	PSD12	26S proteasome non-ATPase regulatory subunit 12	O00232	-1.82
26.	ERN1	Endoplasmic reticulum-to-nucleus signaling 1	O75460	-2.56
27.	FCN3	Ficolin-3	O75636	2.40
28.	HPTR	Haptoglobin-related protein	P00739	2.31
29.	PLMN	Plasminogen	P00747	1.52
30.	ANT3	Antithrombin-III	P01008	-3.10
31.	CO5	Complement C5	P01031	1.70
32.	HBD	Hemoglobin subunit delta	P02042	1.70
33.	CRP	C-reactive protein	P02741	1.63
34.	C1QC	Complement C1q subcomponent subunit C	P02747	2.18
35.	CO9	Complement component C9]	P02748	-2.09
36.	APOH	Apolipoprotein H	P02749	1.72
37.	RET4	Retinol-binding protein	P02753	2.01
38.	AMBP	Protein AMBP	P02760	1.68
39.	PLF4	Platelet factor 4	P02776	-2.02
40.	C4BPA	C4b-binding protein alpha chain	P04003	1.78
41.	A1BG	Alpha-1B-glycoprotein	P04217	1.89
42.	VWF	von Willebrand factor (vWF)	P04275	-2.58
43.	IPSP	Plasma serine protease inhibitor	P05154	-1.92
44.	IC1	Plasma protease C1 inhibitor	P05155	-2.04
45.	F13B	Fibrin-stabilizing factor B subunit	P05160	1.77
46.	CO8G	Complement component C8 gamma chain	P07360	2.07
47.	APOA	Apolipoprotein(a)	P08519	2.13
48.	SAA1	Serum amyloid A-1 protein (SAA)	P0DJI8	-1.91
49.	IGLC3	Immunoglobulin lambda constant 3	P0DOY3	3.19
50.	ITIH2	Inter-alpha-trypsin inhibitor heavy chain H2	P19823	-1.95

51.	DEF3	Neutrophil defensin 3	P59666	-1.94
52.	ACTG	Actin, cytoplasmic 2	P63261	-2.68
53.	HBA	Hemoglobin subunit alpha	P69905	-1.61
54.	FBLN3	EGF-containing fibulin-like extracellular matrix protein 1	Q12805	1.85
55.	TOP3A	DNA topoisomerase 3-alpha	Q13472	-1.90
56.	CUL4B	Cullin-4B	Q13620	-1.98
57.	APOF	Apolipoprotein F (Apo-F)	Q13790	-2.22
58.	HABP2	Hyaluronan-binding protein 2	Q14520	1.92
59.	M3K21	Mitogen-activated protein kinase kinase kinase 21	Q5TCX8	-2.33
60.	CP046	Uncharacterized protein C16orf46	Q6P387	-2.21
61.	MEX3B	RNA-binding protein MEX3B	Q6ZN04	-2.12
62.	CENPU	Centromere protein U	Q71F23	-2.52
63.	MILK2	MICAL-like protein 2	Q8IY33	-2.01
64.	PGRP2	N-acetylmuramoyl-L-alanine amidase	Q96PD5	1.76
65.	CA198	Uncharacterized protein C1orf198	Q9H425	2.25
