# Supplementary material for: Proteomics Profiling of Osteoporosis and Osteopenia Patients and Associated Network Analysis
Source: Int J Mol Sci. 2022 Sep 5;23(17):10200. doi: 10.3390/ijms231710200 (PMC9456664; doi:10.3390/ijms231710200)
Supplement: Supplementary file 1 [file ijms-23-10200-s001.zip › Supplementary Table S2_29.8.2022.pdf]

Supplementary Table S2: The significant and common dysregulated proteins (*n* = 20) among the three groups (control, ON, and OP).

Number	Uniprot ID	Protein Name	Accession Number	Fold Change Ctrl vs ON/OP
1.	IGLC3	Immunoglobulin lambda constant 3	P0DOY3	1.14
2.	TRY1	Trypsin-1	P07477	1.04
3.	M0R1Q1	Complement C3	M0R1Q1	0.87
4.	IGHG2	Immunoglobulin heavy constant gamma 2	P01859	0.85
5.	CA198	Uncharacterized protein C1orf198	Q9H425	0.85
6.	HPTR	Haptoglobin-related protein	P00739	0.83
7.	C1QC	Complement C1q subcomponent subunit C	P02747	0.80
8.	IGHG3	Immunoglobulin heavy constant gamma 3	P01860	0.74
9.	GPX3	Glutathione peroxidase 3	P22352	0.70
10.	HABP2	Hyaluronan-binding protein 2	Q14520	0.70
11.	A1BG	Alpha-1B-glycoprotein	P04217	0.69
12.	CNDP1	Beta-Ala-His dipeptidase	Q96KN2	-0.63
13.	G3P	Glyceraldehyde-3-phosphate dehydrogenase	P04406	-0.66
14.	MILK2	MICAL-like protein 2	Q8IY33	-0.67
15.	MYH14	Myosin-14	Q7Z406	-0.68
16.	M3K21	Mitogen-activated protein kinase kinase kinase 21	Q5TCX8	-0.83
17.	CENPU	Centromere protein U	Q71F23	-0.91
18.	ERN1	Endoplasmic reticulum-to-nucleus signaling 1	O75460	-0.92
19.	ACTG	Actin, cytoplasmic 2	P63261	-0.96
20.	CLN3	Battenin	O95086	-1.00
