# Supplementary material for: Proteomics Profiling of Osteoporosis and Osteopenia Patients and Associated Network Analysis
Source: Int J Mol Sci. 2022 Sep 5;23(17):10200. doi: 10.3390/ijms231710200 (PMC9456664; doi:10.3390/ijms231710200)
Supplement: Supplementary file 1 [file ijms-23-10200-s001.zip › Supplementary Table S3_29.8.2022.pdf]

Supplementary Table S3: The significantly dysregulated proteins ($n = 26$) between the ON and OP groups.

Number	Uniprot ID	Protein Name	Accession Number	Fold Change ON/OP
1	A1BG	Alpha-1B-glycoprotein	P04217	-1.86
2	ACTG	Actin, cytoplasmic 2	P63261	1.77
3	C1QC	Complement C1q subcomponent subunit C	P02747	-1.66
4	CA198	Uncharacterized protein C1orf198	Q9H425	1.79
5	CENPU	Centromere protein U	Q71F23	-1.57
6	CLN3	Battenin	Q13286	1.57
7	CO9	Complement component C9	P02748	1.63
8	CRP	C-reactive protein	P02741	1.66
9	CUL4B	Cullin-4B	Q13620	1.90
10	ERN1	Endoplasmic reticulum-to-nucleus signaling 1	O75460	1.83
11	F13B	Fibrin-stabilizing factor B subunit	P05160	-2.14
12	FBLN1	Fibulin-1	P23142	2.53
13	GPX3	Glutathione peroxidase 3	P22352	-1.54
14	HABP2	Hyaluronan-binding protein 2	Q14520	-1.77
15	HBD	Hemoglobin subunit delta	P02042	-1.66
16	HPTR	Haptoglobin-related protein	P00739	-1.74
17	IGHG2	Immunoglobulin heavy constant gamma 2	P01859	-2.17
18	IGHG3	Immunoglobulin heavy constant gamma 3	P01860	-1.95
19	IGLC1	Immunoglobulin lambda constant 1	P0CG04	-1.61
20	IGLC3	Immunoglobulin lambda constant 3	P0DOY3	-2.67
21	M0R1Q1	Complement C3	M0R1Q1	-1.52
22	MEX3B	RNA-binding protein MEX3B	Q6ZN04	2.56
23	MLXPL	MLX-interacting protein-like	Q9NP71	2.22
24	PROC	Vitamin K-dependent protein C	P04070	2.56
25	SEPP1	Selenoprotein P (SeP)	P49908	1.78
26	TRY1	Trypsin-1	P07477	-1.68
